# Towards a sensitive and accurate interpretation of molecular testing for SARS-CoV-2: a rapid review of 264 studies

**DOI:** 10.2807/1560-7917.ES.2021.26.10.2001134

**Published:** 2021-03-11

**Authors:** Kamelia R. Stanoeva, Annemiek A. van der Eijk, Adam Meijer, Laetitia M. Kortbeek, Marion P.G. Koopmans, Chantal B.E.M. Reusken

**Affiliations:** 1 Center for Infectious Disease Control, National Institute for Public Health and the Environment (RIVM), Bilthoven, the Netherlands; 2 European Public Health Microbiology Training Programme (EUPHEM), European Centre for Disease Prevention and Control (ECDC), Stockholm, Sweden; 3 Department of Viroscience, Erasmus University Medical Center, Rotterdam, the Netherlands; 4 Global Outbreak Alert and Response Network (GOARN), Geneva, Switzerland

**Keywords:** SARS-CoV-2, COVID-19, Laboratory Diagnosis, Polymerase Chain Reaction

## Abstract

**Background:**

Sensitive molecular diagnostics and correct test interpretation are crucial for accurate COVID-19 diagnosis and thereby essential for good clinical practice. Furthermore, they are a key factor in outbreak control where active case finding in combination with isolation and contact tracing are crucial.

**Aim:**

With the objective to inform the public health and laboratory responses to the pandemic, we reviewed current published knowledge on the kinetics of SARS-CoV-2 infection as assessed by RNA molecular detection in a wide range of clinical samples.

**Methods:**

We performed an extensive search on studies published between 1 December 2019 and 15 May 2020, reporting on molecular detection and/or isolation of SARS-CoV-2 in any human laboratory specimen.

**Results:**

We compiled a dataset of 264 studies including 32,515 COVID-19 cases, and additionally aggregated data points (n = 2,777) from sampling of 217 adults with known infection timeline. We summarised data on SARS-CoV-2 detection in the respiratory and gastrointestinal tract, blood, oral fluid, tears, cerebrospinal fluid, peritoneal fluid, semen, vaginal fluid; where provided, we also summarised specific observations on SARS-CoV-2 detection in pregnancy, infancy, children, adolescents and immunocompromised individuals.

**Conclusion:**

Optimal SARS-CoV-2 molecular testing relies on choosing the most appropriate sample type, collected with adequate sampling technique, and with the infection timeline in mind. We outlined knowledge gaps and directions for future well-documented systematic studies.

## Introduction

In 2019 a novel human pathogenic coronavirus, severe acute respiratory syndrome coronavirus 2 (SARS-CoV-2), emerged in Wuhan, China [1], leading to a worldwide outbreak, declared a public health emergency of international concern on 30 January 2020 [2] and a pandemic on 11 March 2020 [3].

SARS-CoV-2 is a positive-stranded RNA virus from the species severe acute respiratory syndrome-related coronavirus, subgenus* Sarbecovirus*, genus *Betacoronavirus*, family *Coronaviridae*. The species contains a wide range of bat and human viruses including SARS-CoV-1 that caused an outbreak in 2002–03. The SARS-CoV-2 origins are still unknown, but zoonotic transmission, with bats (in particular *Rhinolophus* spp.) as the probable primary reservoir and other yet unknown animals as intermediate hosts, is considered the most likely route [4,5].

The disease caused by SARS-CoV-2 is coronavirus disease (COVID-19). In the context of the pandemic, which is currently ongoing, rapid and reliable laboratory diagnosis is essential for detection, confirmation, and ruling out of cases, clinical management and hospital infection prevention measures, source and contact tracing, and (lifting of) isolation measures. Laboratory testing plays a critical role in surveillance to guide public health response. Nucleic acid amplification tests became the first line of testing for SARS-CoV-2 infection recommended by the World Health Organization (WHO) [6]. Serological tests are increasingly being implemented [7,8].

Here, we reviewed the current knowledge on the laboratory aspects of COVID-19 diagnostics with a focus on SARS-CoV-2 molecular assays and summarised key findings for different sample types to support the laboratory response for clinical case management and to inform public health measures to control the pandemic.

## Methods

### Search and selection of published reports

**Figure 1:**
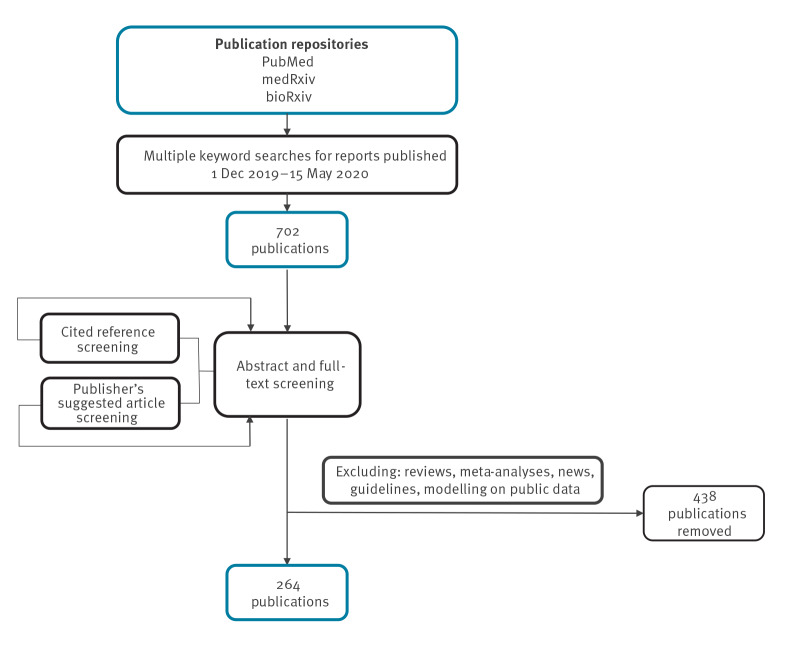
Flow-chart of the review process for publications between 1 December and 15 May 2020 on SARS-CoV-2 molecular detection and/or virus isolation in laboratory specimens

We searched repetitively PubMed, medRxiv and bioRxiv with keyword ‘coronavirus’ limiting to results published between 1 December 2019 and 15 May 2020 and screened the titles and brief descriptions of > 8,700 publications in total. We identified studies on SARS-CoV-2 (including name variations like ‘novel coronavirus’, ‘2019-nCoV’) in humans (all ages), written in English, Chinese or French, and excluded reviews, viewpoints, or news. We selected studies using the words ‘detection’, ‘testing’, ‘PCR’, ‘viral load’, ‘viral kinetics/dynamics/clearance/shedding’, ‘isolation’, ‘persistence’, ‘samples’, ‘bodily fluids’, ‘diagnosis’, ‘case report/series’, ‘case(s)’, ‘cluster’, ‘transmission’, ‘patients’, ‘neonate’, ‘child(ren)’, ‘pregnant’, ‘clinical characteristics/findings/manifestations/features/outcomes’, ‘infection’, ‘pneumonia’, ‘asymptomatic’ in the title, brief description or abstract (if available) aiming to narrow down to clinical reports. This yielded 702 publications for in-depth abstract and full-text screening. Additionally, we scanned literature cited in these articles as well as suggested similar publications and COVID-19 resource collections on the publishers’ websites. Finally, we included 264 studies reporting on SARS-CoV-2 molecular detection and/or virus isolation in any laboratory specimens of COVID-19 cases. We excluded reviews, meta-analyses, news, guidelines, or modelling studies based on public data (Figure 1).

### Data extraction

We aimed to summarise the current information with regard to SARS-CoV-2 infection kinetics in relation to clinical syndrome, in different bodily fluids, while also noting any specifics in some groups (pregnant women, children and immunocompromised individuals).

We extracted data on cases’ demographics such as the number of adults, children, pregnant and immunocompromised individuals as well as age, sex and severity of disease. Moreover information on specimens tested for SARS-CoV-2 was gathered, including the number of patients with collected respiratory (nasopharyngeal/oropharyngeal/other swabs, sputum, bronchoalveolar lavage fluid, endotracheal aspirate), gastrointestinal (GI) (stool, anal/rectal swabs, endoscopic samples) and blood samples, as well as those with oral fluid, tears, urine, cerebrospinal fluid, semen, vaginal fluid, breast milk and perinatal samples. All sample types tested by PCR and viral cultures, where available, were included. The number of patients with sequenced samples was also compiled.

### Definitions of nasopharyngeal swabs and severe disease used for the study

Even though nasopharyngeal swabs are the routine respiratory sample for viral infections, we also included publications with nasal/midturbinate swabs or those using the term ‘nasal’ indiscriminately when describing the nasopharyngeal swab collection technique. 

Due to varying disease severity definitions, we opted for a simplified approach: patients with symptoms or clinical course described as mild, moderate, common, typical, are referred to as patients with ‘mild’ disease, while patients with illnesses characterised as severe, or patients described as critically ill and/or admitted to intensive-care units (ICU), are referred to as patients with ‘severe’ disease. 

### Ethical statement

As the review included publicly available published data, ethical approval was not needed for the study.

## Results

The compiled dataset contained 32,515 COVID-19 cases (Supplementary Dataset). Where possible, we noted duplicate case reports in different publications. Additionally, we aggregated data from 217 adults with data points from 2,777 samples with known collection day post symptom onset (dps).

### Overview of SARS-CoV-2 in different specimens from adults

**Figure 2:**
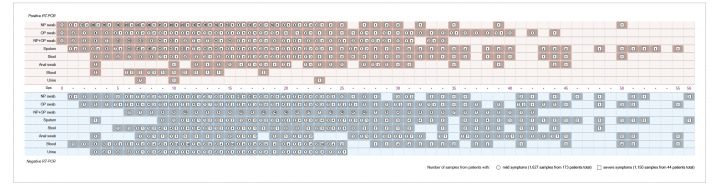
COVID-19 aggregated RT-PCR results in different sample types (n = 2,777) by days post symptom onset in adult patients (n = 217) with mild or severe disease, data from studies published 1 December 2019–15 May 2020

COVID-19: coronavirus disease; dps: days post symptom onset; ICU: intensive care unit; NP: nasopharyngeal; OP: oropharyngeal; RT-PCR: real-time PCR; SARS-CoV-2: severe acute respiratory syndrome coronavirus 2.

**Figure 3:**
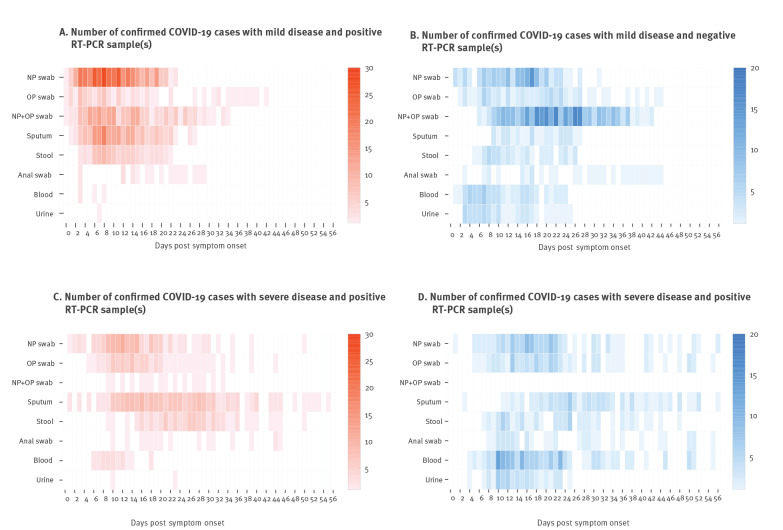
Heatmaps of laboratory-confirmed adult COVID-19 cases with mild and severe disease (n = 217) and with positive and negative RT-PCR results in different sample types (n = 2,777) by days post symptoms onset, data from studies published 1 December 2019–15 May 2020

COVID-19: coronavirus disease; ICU: intensive care unit; NP: nasopharyngeal; OP: oropharyngeal; RT-PCR: real-time PCR.

The distribution of different types of samples testing positive for SARS-CoV-2 as a function of dps and/or illness severity are shown in Figure 2, Figure 3, the Table and the Supplementary Table).

The number of samples with (above dps axis) and without (below dps axis) detectable SARS-CoV-2 RNA are aggregated by sample type, collection dps and disease severity. Sampling for patients with symptoms or clinical course described in the original publications as mild, moderate, common, or typical are shown in circles: a total of 1,627 samples from 173 adults. Sampling for patients with severe disease or who were critically ill and/or admitted to the ICU are shown in squares: a total of 1,150 samples from 44 adults. Asymptomatic patients were excluded. Sample types are as follows: NP swab (NP/midturbinate/nasal swab), OP swab (oropharyngeal/throat swab), NP + OP swab (both swabs collected in one tube or results published aggregated), sputum (induced/spontaneous sputum), stool, anal swab (anal/rectal swab), blood (serum, plasma, whole blood or not specified), urine. Sample collection dps are shown as stated by the authors, and if unavailable extracted from timeline descriptions.

Laboratory-confirmed COVID-19 cases (n = 217) are grouped by disease severity: those (panels A and B) with symptoms or clinical course described in the original publications as mild, moderate, common, or typical (n = 173 adults) are referred to as ‘cases with mild disease’, while those (C and D) with severe disease or critically ill and/or admitted to the ICU (n = 44 adults) are referred to as ‘cases with severe disease’. Asymptomatic patients were excluded. The cumulative number of cases with respective positive (A and C) or negative (B and D) RT-PCR results on different sample types (a total of 1,627 and 1,150 samples from cases with mild and severe disease respectively) on a respective day post symptoms onset are represented using a colour gradient. Sample types are as follows: NP swab (nasopharyngeal/midturbinate/nasal swab), OP swab (oropharyngeal/throat swab), NP + OP swab (both swabs collected in one tube or results published aggregated), sputum (induced/spontaneous sputum), stool, anal swab (anal/rectal swab), blood (serum, plasma, whole blood or not specified), urine. Sample collection by days post symptoms onset are shown as stated by the authors, and if unavailable extracted from timeline descriptions.

**Table:** COVID-19 aggregated RT-PCR results in different sample types (n = 2,777) in adult patients (n = 217) with mild or severe disease, data from studies published 1 December 2019–15 May 2020

Sample type			Cases with positive results			Cases with negative results		
			Number of samples	Number with mild disease	Number with severe disease	Number of samples	Number with mild disease	Number with severe disease
URT	NP swab		461	56	20	254	11	8
	OP swab		151	29	14	165	9	10
	NP + OP swab^a^		197	64	1	296	3	0
	**All URT swabs**		809	149	35	715	23	18
Sputum			376	36	23	131	0	2
GIT		Stool	172	14	11	112	11	11
		Anal swab	34	5	6	71	8	12
		**All GIT samples**	206	19	17	183	19	23
Blood			26	3	6	222	25	24
Urine			3	1	1	106	26	20

COVID-19: coronavirus disease; GIT: gastrointestinal tract; NP: nasopharyngeal/midturbinate/nasal; OP: oropharyngeal/throat; RT-PCR: real-time PCR; URT: upper respiratory tract.

^a ^Both swabs collected in one tube or results published aggregated.

### SARS-CoV-2 kinetics and shedding in the respiratory tract

Some key points found concerning the kinetics and shedding of SARS-CoV-2 in the respiratory tract are summarised in Box 1.

Box 1 **Key points with regards to shedding/kinetics of SARS-CoV-2 in the respiratory tract**

- SARS-CoV-2 replicates in the throat and upper respiratory tract (URT). Respective samples collected early in the infection (regardless of the severity of symptoms) can be used for diagnostic purposes.
- Viral RNA loads peak within the first infection days in the upper and later in the lower respiratory tract; viral load (VL) testing and reporting need to be standardised.
- SARS-CoV-2 RNA is detectable from respiratory samples up to 6 weeks in cases with mild disease and 8 weeks in cases with severe disease, and beyond symptoms’ resolution.
- There were insufficient systematic comparisons between all types of respiratory samples; higher VLs were observed in sputum than in nasopharygeal and oropharyngeal swabs. A more sensitive detection of SARS-CoV-2 detection resulted from nasopharyngeal swabs vs oropharyngeal swabs.
- Computed tomography (CT) findings could sometimes precede viral RNA detection in the URT and full clinical presentation should always be evaluated.

We reviewed 262 studies with respiratory sampling for ≥ 31,957 COVID-19 cases, including data on nasopharyngeal/midturbinate/nasal (NP) swabs (≥ 13,286 cases), oropharyngeal/throat (OP) swabs (≥ 7,301 cases), combined NP and OP swabs (≥ 1,493 cases), sputum (≥ 409 cases), bronchoalveolar lavage fluid (≥ 21 cases), and other respiratory specimens (endotracheal aspirate, bronchoalveolar swab ≥ 21) (Supplementary Dataset) [1,9-268].

#### SARS-CoV-2 isolation from respiratory tract samples

Lower respiratory tract (LRT) bronchoalveolar lavage (BAL) sampling allowed the initial and subsequent virus culture of SARS-CoV-2 [1,11,13]. Almost all BAL specimens described in peer-reviewed literature had detectable viral RNA regardless of sampling timing, disease severity or comorbidities and were useful for ultimate confirmation of difficult cases [11,44,71,77,78].

Virus isolation was successful from NP swabs 2 dps in two cases with mild disease (using Vero E6 cells) [120]; NP and OP swabs 4 dps (using Vero CCL-81 cells) [76] in a mild case [63], and also at 4 dps from NP swab and nasopharyngeal aspirate in another mild case (using Vero E6 cells) [98]. SARS-CoV-2 was isolated from NP swabs of two cases with severe disease 1 and 10 dps [135]. In a German study of nine mild COVID-19 cases, SARS-CoV-2 was isolated up to 8 dps from both URT swabs (16%) and sputum samples (83%) with VL > 10^6^ copies/mL. Furthermore, the authors detected viral subgenomic mRNAs (sgRNA) which led them to conclude there was ongoing viral replication in the throat up to 5 dps. Sequencing data also showed the continuous presence of two genotypes of SARS-CoV-2 differing by a single mutation in the throat and lungs samples of a patient [131]. 

La Scola and colleagues cultured 174 NP swabs and nine sputum samples testing positive via PCR (from 155 patients total) and succeeded with virus isolation from 129 samples (124 with observable cytopathic effect on Vero E6 cells). They observed a strong correlation between cycle threshold (Ct) values and virus isolation: 100% isolation rates from samples with a Ct of 13–17 decreasing to 12% at Ct 33 and no isolation from samples with Ct ≥ 34 [211]. 

An Indian study was successful in isolating SARS-CoV-2 (using Vero CCL-81 cells) from respiratory samples in nine of 12 samples with Ct values ranging 16–25.1 [217].

#### SARS-CoV-2 detection with regards to the infection timeline and disease severity

Despite the growing amount of literature, only two studies documented NP swabs, OP swabs, and sputum, collected sequentially in the same 16 [172] and 49 [240] patients respectively, while two studies described an upper respiratory specimen paired with sputum for a total of 11 cases [39,131]. SARS-CoV-2 RNA was detected in NP and OP swabs from symptoms onset up to 42 dps in cases with mild disease [158] and 50 dps in those with severe disease [172]. Sputum yielded positive results up to 27 dps in cases with mild disease [131], but 55 dps in those severely ill [172] (Figure 2 and Figure 3). Sun and colleagues aggregated data on 175 NP swabs, 88 OP swabs, and 62 sputum samples, and estimated a median/95^th^ percentile time until loss of detection as follows: NP 22.7/46.3 dps, OP 15.6/32.8 dps, and sputum 20/43.7 dps for 43 cases with mild disease vs NP 33.5/49.4 dps, OP 33.9/53.9 dps, and sputum 30.9/44.7 dps for six cases with severe disease [240].

Nevertheless, we also aggregated the data from 31 observational studies with available infection timeline and respiratory sampling for 216 cases [9,18,23,25,29,34,37,39,42-45,58,59,63,72,85,98,111,116,120,131,134,158,160,171,172,176,187,197,206] to provide a summary (Figure 2, Figure 3, Table, and Supplementary Table).

Several studies reported SARS-CoV-2 RNA detection from the URT for a median period of 10–20 days [53,59,79,99,105,187,191] with a prolonged detection observed in cases with severe disease [99,105,191]. An aggregation of retrospective observations from 191 hospitalised adults in Wuhan showed a median duration of URT viral RNA detection of 20 (interquartile range (IQR): 16–23) dps with continuous detection until death in non-survivors and ranges 8–37 dps in survivors [79]. SARS-CoV-2 RNA was detectable in URT samples well beyond waning of respiratory symptoms [37,46,107,131].

A study in Nanchang (21 cases with mild, 10 with severe disease) observed clearance in NP swabs within 10 dps in 90% of the cases with mild disease compared with continuous RNA detection > 10 dps in all cases with severe disease [99]. Feng and colleagues detected SARS-CoV-2 RNA in NP swabs of 24 cases with mild disease for 16 ± 7 days compared with 22 ± 4 days in eight ICU patients [105].

SARS-CoV-2 remained detectable in OP swabs ≥ 2 weeks, (nine cases with mild disease) and in sputum > 3 weeks (six cases with mild disease) despite symptoms resolution [131]. Chen and colleagues described recurring SARS-CoV-2 RNA positivity in OP swabs of a patient until 30 dps (VL: 4.56×10^2^ copies/mL), well after pneumonia resolution and hospital discharge [37]. Prolonged viral RNA detection in OP swabs (range: 5–30 dps for 22 cases) was also reported for cases with mild disease regardless of symptoms, including > 3 weeks in eight cases [199]. The study did not address if infectious virus could be detected. Another study including 66 COVID-19 cases found a median of 9.5 (6–11) dps until the first negative results in OP swabs [53]. In some cases, such as in a total of five patients with mild symptoms in Wuhan, SARS-CoV-2 RNA was re-detected 4–15 days following a last negative OP swab and hospital discharge [104]. As reported by the authors of the study [104], the COVID-19 reactivation could have been due to prolonged disease course too. Another Wuhan-based study reported re-hospitalisation with mild symptoms for 11 cases within a median of 16 (range: 6–27) days post initial hospital discharge, of whom four had viral RNA detected in OP swabs [254].

#### SARS-CoV-2 RNA detection considering health condition and demographic characteristics

The duration of SARS-CoV-2 shedding may be related to patient’s general health condition. In a Wuhan-based study, 27 of 56 cases with mild disease had prolonged SARS-CoV-2 RNA detection > 24 days in NP/OP swabs, associated with old age and comorbidities. The proportion of positive respiratory samples decreased from 89% to 66%, 32%, 5%, and 0% in weeks 2–6 since symptoms onset [187]. Xu and colleagues summarised respiratory samples’ data from 113 patients and observed a median time of 17 (IQR: 13–22) dps where RNA could be detected. Prolonged detection ≥ 15 dps was associated with males, old age, hypertension, severe illness upon admission, invasive mechanical ventilation, and corticosteroid treatment [150]. A Wuhan-based study on 41 discharged cases who had had severe disease reported SARS-CoV-2 RNA detection in OP swabs for a median of 31 (IQR: 24–40, range: 18–48) dps and no significant difference between male and female sex, nor between cases < 65 and ≥ 65 years of age [186].

#### Viral load in respiratory specimens in the context of disease severity, infection timeline and demographic characteristics

In the studies that provide quantitative results (n = 26) [9,10,25-27,34,37,39,40,59,63,99,111,119,120,122,131,158,160,161,170,172,202,206,220,248], the highest VL in URT specimens were reported in the early days of the disease [34,39,59,63,99,120,131,170,172], also before development of respiratory symptoms [39,119,170]. A study of nine cases with mild disease reported SARS-CoV-2 RNA detection from all NP and OP swabs in the first 5 dps with (average VL: 6.76×10^5^ copies/swab and maximum 7.11×10^8^ copies/swab), whereas detection rate in subsequent swabs was only 40% up to 28 dps (average VL: 3.44×10^5^ copies/swab) [131].

Among four patients (two with mild, two with severe disease), VLs in NP swabs ranged from 7.4 log_10_ copies/1,000 cells (mild case at 2 dps) and 7.1 log_10_ copies/1,000 cells (case with severe disease at 6 dps) to negative at 9–14 dps, while for a critically ill case they were in the range of 4.4–6.7 log_10_ copies/1,000 cells and persisted 24 dps until death [120]. Among 61 healthcare workers, VLs of self-collected combined NP and OP swabs were significantly lower for 56 asymptomatic vs five symptomatic cases [248]. A study on 12 cases (nine with mild, three with severe disease) showed that Ct values from respiratory samples were correlated with disease severity scores like acute respiratory distress syndrome (ARDS) index PaO_2_/FiO_2_ ratio and lung injury Murray score, as well with biochemical indicators like albumin levels and lymphocytes and neutrophils percentages, and concluded VLs could serve as a COVID-19 severity predictor [17]. 

In a paediatric dialysis unit, where 12 COVID-19 cases (six asymptomatic, six mild), mainly healthcare workers, had had a NP swab, the VLs were significantly higher for the patients with symptoms [212]. Kimball and colleagues reported no significant difference between the Ct values in NP swabs collected from 10 symptomatic, 10 presymptomatic and three asymptomatic cases in a nursing facility [119].

He and colleagues reported high VL for 414 OP swabs from 94 patients in the early days of infection and gradual decrease until ca 21 dps with no difference when stratified by sex, age, or disease severity [170]. However, VLs in the sputum of 22 cases with mild disease reached a peak in week 2 after symptoms onset and were significantly lower than those of 74 cases with severe disease [191]. In a cohort of 92 cases (51 with mild disease, 11 with mild disease turning severe, 30 with severe disease) low Ct values (high VL) in sputum were correlated with severe COVID-19 and risk of progression to severe disease [202].

Comparing respiratory specimens, higher VL were reported for sputum than respiratory swabs [9,40,122,131,172]. A study using RT-PCR and droplet digital PCR found both significantly higher positive rates and average VL in sputum (66% and 17,429 ± 6,920 copies/test) compared with OP swabs (37% and 2,552 ± 1,965 copies/test) and NP swabs (16% and 651 ± 501 copies/test) [122]. For 16 critically ill patients, sputum and endotracheal aspirate samples all had detectable SARS-CoV-2 RNA at levels significantly higher than NP and OP swabs with positivity rates of 81% and 63% respectively [172]. In nine cases with mild disease up to 5 dps, the maximum SARS-CoV-2 VL in sputum (2.35×10^9^ copies/mL) was higher compared with respiratory swabs (7.11×10^8^ copies/swab). However, examining paired sputum and swab samples 2–4 dps in seven patients showed higher virus concentration in swabs (two cases), sputum (two cases), and similar virus concentrations in both for the remaining five cases [131]. In a cohort of 52 patients, the positivity rates of sputum samples (77%) were higher than OP swabs (44%) with a significant difference when comparing cases with positive sputum sample and negative OP swab (40%) vs those with negative sputum and positive OP swab (8%) [180].

#### Choice of a respiratory specimen for SARS-CoV-2 diagnostic

Choosing the most appropriate respiratory specimen depends on timing in the infection course. However, well-documented studies comparing all respiratory and other sample types collected in a known timeline were limited. A preprint study in Guangdong on 866 respiratory samples from 213 symptomatic cases (37 with severe disease), showed that apart from BAL, sputum is the most sensitive sample type for COVID-19 laboratory diagnosis, followed by NP swabs [26]. In the period 0–7 dps, the highest positivity rate was observed in sputum (89% of cases with severe disease, 82% of cases with mild disease) followed by NP swabs (73%, 72%) and OP swabs (60%, 61%). In the period 8–14 dps the same order in positivity rates was observed: sputum (83% of cases with severe disease, 74% of cases with mild disease), NP swabs (72%, 54%), and OP swabs (50%, 30%); as for ≥ 15 dps: sputum (61%, 43%), NP swabs (50%, 55%), and OP swabs (37%, 11%). Sputum samples 0–7 dps also yielded the lowest median Ct values (25 in cases with severe disease, 28.5 in cases with mild disease). BAL samples 8–14 dps were positive for SARS-CoV-2 RNA in 12 cases with severe disease and negative for three cases with mild disease. Beyond 15 dps the study showed 79% positivity in BAL in cases with severe disease [26].

Further peer-reviewed data was also in favour of sputum, or NP swabs [71,77]. A retrospective study of 4,880 cases in Wuhan found 38% positive rate for 4,818 NP and OP swabs compared with 49% for 57 sputum specimens and 80% for five BAL [71]. Another Chinese study including 205 COVID-19 cases yielded overall 1,070 samples with the following positivity rates: 93% for 15 BAL samples, 72% for 104 sputum specimens, 63% for eight NP swabs, 46% for 13 fibrobronchoscope brush biopsy samples, 32% for 398 OP swabs, 29% for 153 faeces samples, 1% for 107 blood samples [77]. Only BAL specimen sequencing, and BAL and sputum samples PCR could confirm SARS-CoV-2 co-infection with influenza A in an ICU patient, whereas repeated NP swabs were negative [78]. Although sputum might seem like a sample of choice, it was described that only a third of 1,099 COVID-19 patients had a productive cough [50], suggesting that in practice NP swabs would be preferable in most cases. Higher sensitivity for NP swabs in comparison to OP swabs was observed as well in other studies and case reports [34,52]. A study with sequential sampling in 18 patients (72 NP and 72 OP swabs) showed higher VL in the nose than the throat [34].

SARS-CoV-2 shedding potential in asymptomatic and pre-symptomatic individuals needs to be elucidated [74,94,101,119,139,144,155,156,160,167,170,224,227,269,270]. Multiple studies worldwide reported SARS-CoV-2 RNA detection in respiratory samples from cases with epidemiological link and no (189 adults, 29 children) [9,31,34,36,61,70,74,94,116,119,123,139,144,155,160,199,205,214,224,227,248] or mild/non-specific symptoms (23 adults, one child) [9,12,15,18,20,24,25,28,30,39,61,74,116,227]. One described SARS-CoV-2 RNA detection in OP swabs for 17 days in an otherwise asymptomatic patient [70]. In a series of testing campaigns in Iceland that led to SARS-CoV-2 RNA detection in combined NP and OP swabs of a total 1,321 cases, those reporting any symptoms ranged between 46% (random population screening) and 94% (targeted screening) [167].

At this stage, it was unclear whether SARS-CoV-2 affects the upper or the lower respiratory tract first, or maintains independent replication in both sites. Thus, choosing between NP and OP swabs, or sputum as sampling strategy should be done with the purpose (general population screening or confirmation of suspected cases) and potential infection timeline in mind. URT sampling would be preferable in the early infection days, especially in asymptomatic or mildly symptomatic suspected cases, whereas lower respiratory sampling provides more reliable confirmation in advanced COVID-19 with lungs’ involvement. Cases with an epidemiological link, radiological findings, and an initial negative result should be monitored further by PCR and evaluated in conjunction with their clinical presentation [10,22,35,38,41,55,56].

The discrepancy between URT and LRT test results triggered a discussion about the lack of sensitivity of PCR testing. In a study among 1,014 patients in Wuhan 59% (95% confidence interval (CI): 56–62%) of OP swabs were positive whereas 88% (95%CI: 86–90%) had chest CT findings within a median of 1 day of the PCR test, consistent with an earlier resolution of viral replication in URT than in LRT samples. Furthermore, 308 patients (75%) had negative PCR results in conjunction with radiologic findings and 14 of 15 cases with CT findings tested positive on a follow-up PCR within a mean of 5 days, a finding that may be more difficult to explain [41]. Another study that aggregated data on PCR results and CT imaging showed all 167 patients had a positive OP swab by the end of their hospitalisation [22]. A multicentre study of 80 COVID-19 cases (no critically ill) in Jiangsu reported the following positivity rates in repeated OP and/or NP swabs collection until laboratory confirmation of the infection: 51%, 38%, and 11% upon the first to third test PCR test [56]. Another Chinese, Hubei-based, study reported SARS-CoV-2 RNA detection in OP swabs of 74%, 12% and 14% of 91 cases upon the first to third test and no significant difference between the 30 cases with severe disease and 61 cases with mild diseases included [221]. A New York-based study on 5,700 COVID-19 cases (including 1,281 in ICU) reported detection upon first NP swab test in 97% (n = 5,517) of cases compared with 3% (n = 183) with repeated tests [193].

### SARS-CoV-2 shedding in the gastrointestinal tract

Some key points found about shedding of SARS-CoV-2 in the GI tract are summarised in Box 2.

Box 2 **Key points with regards to gastrointestinal shedding of SARS-CoV-2**

- SARS-CoV-2 can be isolated from faeces and RNA can be detected regardless of GI symptoms.
- Faecal sampling is not recommended for diagnostic screening (except for laboratory diagnosis of suspected cases with negative respiratory tract results).
- Prolonged GI viral RNA detection can occur up to 7 weeks from either symptoms onset or hospitalisation, well after respiratory tract clearance and symptoms resolution in some patients.

We reviewed 47 studies providing data on GI sampling in ≥ 629 COVID-19 patients, including stool specimens (≥ 486 cases), anal/rectal swabs (≥ 198), and others (endoscopic samples, n = 14) (Supplementary Dataset) [9,27,39,40,42,43,48-51,53,58-60,63,64,77,84-86,89,91,93,100,102,105,109,117,120,121,126,131,137,152,158,168,171,172,178,179,191,203,206,229,237,240,271].

#### SARS-CoV-2 isolation from gastrointestinal samples in regard of the infection timeline

SARS-CoV-2 was isolated from stool samples 15 dps from a COVID-19 patient with severe pneumonia (using Vero cells) [271] and from two patients without diarrhoea [77]. In a study involving nine cases with mild disease, virus isolation (on Vero E6 cells) was unsuccessful in stool samples 6–12 dps from four patients, and no virus replication evidence was found through sgRNA assays despite detectable high VL [131]. SARS-CoV-2 nucleocapsid protein was detected in the cytoplasm of gastric, duodenal, and rectum glandular epithelial cells in one patient [58]. The gastric fluid samples of six of 13 critically ill patients were positive for SARS-CoV-2 RNA [172].

#### SARS-CoV-2 detection and presence/absence of gastrointestinal or other symptoms

GI disease has been described for some COVID-19 patients [9,10,12,14,16,63,83,110,272] and was one of the clinical signs associated with a positive SARS-CoV-2 test [272]. Though there was evidence of SARS-CoV-2 RNA detection in the GI tract, it was not necessarily in cases with GI symptoms [48,102]. Examining studies with an available timeline of sampling (Figure 2, Figure 3, Table, and Supplementary Table), SARS-CoV-2 RNA detection was reported between 3 and 50 dps in stool samples of 14 cases with mild [59,63,120,131] and 11 cases with severe disease [58,59,172], regardless of the presence of diarrhoea. Anal swabs had detectable SARS-CoV-2 RNA between 3 and 45 dps in six cases with mild disease [158,171,206] and seven cases with severe illness [42,172]. No systematic comparison between viral detection in stool and anal swabs was available.

Prolonged GI SARS-CoV-2 RNA detection after the resolution of respiratory symptoms and/or convalescence was observed in several studies [53,58,60,102,131,137,191], though infectious virus shedding is still an outstanding question. In a German study, stool samples remained RNA positive ≥ 21 dps for six cases with mild disease, including a patient with, as suggested by the authors, potential independent intestinal tract replication, based on comparison with the SARS-CoV-2 URT kinetics [131].

#### SARS-CoV-2 detection in stool compared to other types of specimens

Zheng and colleagues reported a 59% positivity rate in 842 stool samples from 96 patients and a median viral RNA detection duration of 22 (IQR: 17–31) dps that was significantly longer than in sputum/saliva and serum samples [191]. In a Chinese study on 98 COVID-19 cases (18 with severe disease), paired OP swabs and stool specimens for 74 cases yielded the following results: 41 with SARS-CoV-2 detection in stool for a mean 27.9 dps (standard deviation (SD): 10.7) that was 11 days after the clearance in OP swabs with mean detection 16.7 dps (SD: 6.7); while the remaining 33 patients with negative stool results had positive OP swabs for a mean 15.4 dps (SD: 6.7). A patient had detectable SARS-CoV-2 RNA in stool 47 dps and another for 33 days after respiratory clearance. Disease severity was not associated with prolonged GI shedding in this cohort [102]. In another cohort of 42 patients (11 cases with severe disease) with GI symptoms in China, 28 cases (nine with severe disease) had a median RNA detection period of 11 (IQR: 7–13) dps until first positive stool sample compared with 6.5 (IQR: 3‐7.25) dps for OP swabs. More than half (n = 18, five with severe disease) remained stool positive for a median 7 (IQR: 6‐10) days after negative OP swabs [137]. A total of 39 (53%) of 73 hospitalised patients had detectable SARS-CoV-2 RNA in stool and for 17 patients (20%), stools remained positive after respiratory samples turned negative [58]. Another Chinese study on recovering patients (n = 55) found a median of 11 (9–16) dps until the first negative results in stool: 43 patients had a 2 (1–4) days median delay in clearance in faeces compared with OP swabs, while in 12 patients both turned negative at the same time [53]. Sun and colleagues aggregated data on 165 stool samples and estimated a median/95^th^ percentile time until loss of detection of 24.5/45.6 dps for 43 cases with mild disease and 32.5/48.9 dps for six cases with severe disease, both comparable to the estimates for NP swabs [240].

#### SARS-CoV-2 detection in anal swabs

Like observations for stool specimens, prolonged SARS-CoV-2 RNA detection was reported for anal swabs [42,70,158,168,171,172,206]. Presence of SARS-CoV-2 RNA in anal swabs seemed linked to disease severity in a Guangzhou cohort (two cases with severe and 16 with mild disease) [9]. Zhang and colleagues found positive anal swabs in four of 16 patients upon hospitalisation and six of 16 cases at day 5 of hospitalisation [27]. A Chinese study with discharge criteria of two consecutive negative OP swab and a negative anal swab, reported a median RNA detection duration of 12 (IQR: 9–14; range: 4–34) days between the first detection and two (consecutive) negative samples for 24 patients [158]. SARS-CoV-2 RNA detection in anal swabs ≥ 17 days was observed in an asymptomatic patient [70].

#### Frequency of patients with viral shedding in the gastrointestinal tract and viral loads 

Large well-documented cohort studies are needed to estimate the proportion of COVID-19 cases with continuous GI shedding and the SARS-CoV-2 VL levels over time. A 29% SARS-CoV-2 RNA positivity rate in stool samples was observed in a study aggregating data on 205 patients (unclear how many provided the analysed 153 stool samples) [77]. Few studies (n = 10) provided quantitative data on SARS-CoV-2 RNA detection in stool and anal swabs for 38 COVID-19 cases [27,59,63,131,158,168,171,172,206]. SARS-CoV-2 GI VL in adults seemed to be subjectively lower (higher Ct values) than in the respiratory tract in 25 cases [59,63,131,168,171,172,206], and higher (lower Ct values) in four cases with mild disease [131,168,171], though meaningful conclusions cannot be drawn from such small sample sizes and non-systematic observations. There was no significant difference between the VL in stool between 22 cases with mild and 71 with severe disease [191].

### SARS-CoV-2 detection in blood

Some key points found about SARS-CoV-2 detected in blood are summarised in Box 3.

Box 3 **Key points with regards to SARS-CoV-2 detection in blood**

- Blood sampling is not recommended for initial diagnostics.
- There is no evidence of SARS-CoV-2 isolation from blood.
- SARS-CoV-2 RNA detection in blood could be a sign of severe disease up to 4 weeks post symptoms onset and could be used as a clinical monitoring tool.

We reviewed 32 studies providing blood samples (whole blood, plasma or serum) data of ≥ 389 COVID-19 patients (Supplementary Dataset) [9,10,27,39,42,48,49,51,53,58,59,63,64,77,89,105,109,118,120,122,131,133,134,145,155,172,178,182,191,196,206,223]. A systematic comparison of SARS-CoV-2 RNA detection in different types of blood samples was lacking. No virus isolation from blood samples was reported.

#### SARS-CoV-2 detection with regard to the infection timeline and disease severity

Summarising data on COVID-19 patients with known infection timeline (Figure 2, Figure 3, Table, and Supplementary Table), SARS-CoV-2 RNA was detected 3–18 dps in 14 patients: 18 blood samples of 10 cases with severe disease [9,42,58,59,172] and four samples of four cases with mild disease respectively [9,39,206]. SARS-CoV-2 RNA presence in blood was linked with disease severity [42] and reported in further 54 cases with severe disease [10,27,105,109,120,172,182,191]. Additionally, viral RNA was detected in blood samples from 35 cases with mild disease [10,27,105,109,178,191,206], one asymptomatic infant [51] and three samples from unspecified cases [77]. SARS-CoV-2 RNA detection in blood might be useful as a laboratory sign of deterioration in cases with severe disease. SARS-CoV-2 RNA was detected in blood samples from 16 cases with mild disease for 10 ± 6 days and seven ICU patients for 15 ± 6 days [105]. A Chinese study reported a median viral RNA detection duration in the serum of 16 (IQR: 11–21) dps, and 27% serum positivity rates in 22 cases with mild disease compared with 45% in 74 cases with severe disease. Detection rates peaked in weeks 2–3 since symptoms onset in all patients with detectable SARS-CoV-2 RNA (17 with severe and three with mild disease) and dropped to 11% (n = 5) for cases with severe disease and 0 for cases with mild disease in week 4. However, Ct values had no significant difference between cases with severe and mild disease [191].

#### SARS-CoV-2 detection in blood compared to other types of specimens

A patient in critical condition had lower, but detectable SARS-CoV-2 RNA in plasma (Ct: 35.8–38.4; 7–12 dps) compared with NP swabs (6.7–4.4 log_10_ copies/1,000 cells; 7–24 dps) [120]. None of nine adults diagnosed with COVID-19 using OP swabs had detectable viral RNA in blood when tested with three different kits [48]. Although SARS-CoV-2 was detected and successfully isolated from respiratory samples, all 31 serum samples from nine cases with mild disease tested negative [131]. Finally, none of the serum samples from 14 convalescent patients (no respiratory symptoms and two consecutive negative OP swabs) were positive for SARS-CoV-2 RNA despite simultaneous detection in OP swabs and stool [53].

### Other specimens: oral fluid, tears, urine, cerebrospinal fluid, peritoneal fluid, semen

Some key points found on SARS-CoV-2 in various other types of specimens than the ones previously considered are presented in Box 4.

Box 4 **Key points with regards to SARS-CoV-2 in semen, tears, urine and oral, cerebrospinal or peritoneal fluid**

- Self-collected oral fluid/saliva could be used as an alternative to respiratory sampling.
- SARS-CoV-2 RNA is detectable in oral fluid/saliva up to 4 weeks.
- RNA is rarely detected and SARS-CoV-2 has not been isolated in conjunctival secretions.
- There is limited SARS-CoV-2 RNA detection in urine and no virus isolation.
- SARS-CoV-2 can be visualised in brain tissue post mortem and viral RNA has been detected in cerebrospinal fluid.
- SARS-CoV-2 RNA has been detected in semen.

#### SARS-CoV-2 detection in oral fluid and saliva

Fifteen studies reported on oral fluid sampling (with varying collection methods) in > 339 COVID-19 cases [21,62,89,97,105,109,148,164,178,190,191,194,208,209,265]. No study compared the different collection methods, e.g. self-collection, sampling by a healthcare worker, swabbing, stimulated secretion. Self-collected deep throat (posterior oropharyngeal) saliva was suggested as an alternative to sputum and yielded positive PCR results in 11 of 12 hospitalised COVID-19 patients in Hong Kong, as well as three positive and two negative virus cultures [21].

Further 23 patients with 173 saliva and endotracheal aspirate samples, studied by the same group, had median VL of 5.2 log_10_ copies/mL (IQR: 4.1–7.0) at presentation. The highest saliva VL were reported in week 1 from symptoms onset (20 patients), followed by a gradual decline, and prolonged RNA detection ≥ 20 days (seven patients) [109]. In a summary of the first cases in Hong Kong VLs in saliva were reported as 5.9×10^6^ copies/mL compared with 3.3×10^6^copies/mL in combined NP and OP swabs [62]. An Australian study screened 522 paired saliva and NP swabs and detected SARS-CoV-2 RNA in 33 saliva samples of 39 cases confirmed by NP swab. VLs were lower in saliva compared with NP swab with both sample types positive up to 21 dps.

Among 50 subjects with negative NP swab, one had a positive saliva sample [190]. Similarly, a Thai study screened 200 participants with paired saliva and combined NP and OP swabs and found 16 cases with matched positive saliva and swabs, two with only saliva positive and three with only NP + OP swab positive. VLs were comparable and a 97.5% agreement was observed between saliva and combined NP + OP swabs [265]. A pre-print study in the United States (later published in the New England Journal of Medicine [273]) including 44 cases reported comparable/superior sensitivity of saliva to NP swabs and higher SARS-CoV-2 saliva VL for 38 matched samples [194]. In another pre-print study saliva was collected from 31 patients (26 mild, five cases with severe disease) after stimulation of the salivary gland, paired with OP swab, and tested positive for SARS-CoV-2 RNA in four (three with severe, one with mild disease) of 13 cases with positive OP swab [97]. A study in Zhejiang confirmed COVID-19 in 96 patients by testing 668 sputum and 1,178 saliva samples but did not specify the positivity rates of the samples types separately. Taken together the latter declined from 95 to 54% in weeks 1–4 since symptoms onset with a median RNA detection duration of 18 (IQR: 13–29) days [191]. In 25 cases SARS-CoV-2 RNA detection in the saliva was reported for 13 ± 5 days in cases with mild disease and 16.5 ± 6 days in ICU patients [105]. A mild case in Wuhan had SARS-CoV-2 RNA detectable in OP swabs, saliva (Ct = 18.7), and urine sediment 54 dps, and continuous detection in OP swabs over 70 dps [208]. SARS-CoV-2 was detected in all saliva samples collected by drooling technique from 25 cases with severe disease [164], including two patients with same-day negative respiratory sampling in NP and bronchoalveolar swabs [164,209]. A comparison between throat wash with saline solution and NP swabs collected 48–57 dps in 11 cases found inconsistent results and higher positivity rates in throat wash [148].

#### SARS-CoV-2 detection in conjunctival secretions and tears

We reviewed six studies reporting conjunctival swab sampling in 137 COVID-19 cases [45,105,111,120,130,172]. SARS-CoV-2 RNA was detected, but virus not isolated, in the tears and conjunctival secretions of one mildly symptomatic patient with conjunctivitis [45], while samples and cultures from 46 patients without ocular symptoms were negative [45,111]. SARS-CoV-2 RNA detection in tears was also reported for one critically ill patient [172] and in five cases with unspecified disease severity [105]. Two cases with severe disease of 12 COVID-19 patients with ocular symptoms had positive conjunctival swabs [130]. No SARS-CoV-2 was detected in conjunctival swabs of four cases, including one with severe disease and conjunctivitis [120].

#### SARS-CoV-2 detection in urine

We reviewed 31 studies featuring urine samples from ≥ 369 patients [9,39,40,43,48,49,51,53,58,59,63,64,77,89,105,109,120,122,129,131,133,137,172,178,191,197,206,208,223,237,274]. None reported SARS-CoV-2 isolation from urine. A letter published shortly after the cut-off date of this review (not included in Supplementary Dataset) described successful isolation of SARS-CoV-2 (on Vero E6 cells) at 12 dps in a case with severe disease [275]. SARS-CoV-2 RNA was only detected in the urine of four patients (three patients with a positive sample upon OP swab turning negative) [53], including one woman with positive OP swab at 7 dps [206], one critically ill patient with suspected systemic COVID-19 infection [172], one case with urine abnormalities who later developed severe COVID-19 [274], and one neonate with mild infection 6–17 dps [178]. Urine sediments were positive for SARS-CoV-2 RNA in five COVID-19 cases [129,208].

#### SARS-CoV-2 detection in cerebrospinal fluid

Cerebrospinal fluid (CSF) sampling for a total of 14 patients was reported in eight studies [128,138,185,189,222,230,252,257]. SARS-CoV-2 presence in the brain was evidenced post-mortem in a case who had had severe disease: viral particles were observed in the frontal lobe, moreover tissues samples, but not CSF, had detectable viral RNA [189]. SARS-CoV-2 RNA was detected (Ct > 36 for nucleocapsid protein (N) target only in a N/N2-based Japanese assay) in the CSF of a patient with meningitis 9 dps [138]. However, viral RNA was not detectable in CSF of six patients with Guillain–Barré syndrome [185,252], nor of two patients with mild respiratory symptoms and suspected viral meningoencephalitis [230], nor of two patients with intracranial haemorrhage and positive NP swabs [222], nor of a child with Kawasaki-like disease [257]. CSF was not tested in a patient with suspected COVID-19 related acute necrotising encephalopathy [128], nor in another two with Guillain–Barré syndrome [174,244].

#### SARS-CoV-2 detection in peritoneal fluid

SARS-CoV-2 was not detected in peritoneal fluid samples collected during an appendectomy in a patient without respiratory symptoms, but a positive NP swab [242].

#### SARS-CoV-2 detection in semen

We reviewed five studies reporting on semen testing in 102 COVID-19 cases [181,197,235,267,268]. SARS-CoV-2 RNA was detected in semen from six (four with acute COVID-19 and two recovering) of 38 patients [235]. SARS-CoV-2 was not detected in semen collected during the recovery of 64 cases (nine convalescent cases with severe disease, 54 with mild disease and one asymptomatic case) [181,197,267,268], nor in the testis samples from a deceased case who had had severe disease [181].

### Pregnancy and infancy, vaginal sampling

We reviewed 30 studies including 400 pregnant women and their infants [19,47,54,57,64,66,81,84,92,106,110,115,118,127,146,159,162,163,173,179,201,204,210,213,223,225,226,231,234,255]. No confirmed mother-to-child transmission was reported for these babies delivered mainly via caesarean (C)-section. A single infant, delivered via C-section, isolated, and formula-fed, had a positive pharyngeal swab 36 hours after delivery. Cord blood and placenta tested negative, but it was uncertain whether it was a vertical transmission or nosocomial infection [84]. Similarly, another infant delivered via C-section and quarantined, had five NP swabs collected until 16 days of age negative for SARS-CoV-2 RNA, but serology suggestive of in utero infection. Amniotic fluid or placenta were not tested and vertical transmission could not be confirmed/excluded [115]. Buonsenso and colleagues reported on a single case of SARS-CoV-2 RNA detectable in placenta, umbilical cord blood and three of five breast milk samples collected in the first 5 days from delivery via C-section. The infant was fed with expressed breast milk that tested negative on days 14–17 and his NP swabs were negative on days 1, 3, 18 as well as the OP and anal swabs on day 18 [225]. An infant and her mother were diagnosed with COVID-19 using NP swabs on day 7 post-delivery via C-section. The infant had negative NP swabs after 14 days and horizontal transmission was considered most likely [238]. Altogether SARS-CoV-2 RNA was not detected in 41 amniotic fluid [19,54,64,92,162,179,201,223], nor in 41 umbilical cord blood [19,54,64,84,92,162,201,225], nor in 10 placental tissue samples [54,57,64,84,92,223,225]. It is unclear whether SARS-CoV-2 could be transmitted through breastmilk, so far it was not detected in samples from 39 mothers [19,51,64,84,91,92,162,178,201,225,226,237,238].

It is unclear whether shedding occurs in the female reproductive systems posing risks for vaginal delivery and sexual intercourse. In a study of 35 cases with mild disease with an age range of 37–88 years, viral RNA was not detected in exfoliated cervical cells nor vaginal fluid [226]. Vaginal fluid and vaginal swabs in further 22 women also tested negative [92,115,133,179,201,223,237] including in 10 with severe disease [133].

### Children and adolescents

We reviewed 64 studies describing COVID-19 in 1,510 children and adolescents [9,17,31,32,45,49,51,56,68,74,83,84,86,87,89,91,93-95,100,101,106,108,112,114,115,121,126,140,143-145,155,156,160,166,168,177,178,183,184,193,199,203,205,212,214,225,227,229,232,233,236-238,243,246,247,249-251,253,257,264]. Some further studies included cases < 18 years old grouped with adults and/or provided incomplete stratification by age (Supplementary Dataset).

In a study of 36 cases with mild disease of ages 1–16 years it took a mean of 10 (range: 7–22) days until the first negative results in NP swabs [114]. An asymptomatic 6-month infant maintained detectable SARS-CoV-2 RNA in NP swabs until 17 days of hospitalisation and had a positive stool sample on day 9 [51]. In an Italian study, 168 paediatric cases (four asymptomatic, 131 with mild disease, 33 with severe disease) nationwide were diagnosed via NP/OP swabs [233]. Verdoni and colleagues reported an increase of Kawasaki-like disease cases in March–April 2020 with two of 10 children with positive NP swabs and eight with positive serology [257].

A case-report documented an episode of detectable SARS-CoV-2 RNA in blood and transient fever in an otherwise asymptomatic 6-month infant [51]. 

Prolonged GI SARS-CoV-2 RNA detection after respiratory samples clearance was observed in stool samples up to 35 dps for 20 children aged 0.15–10 years [49,89,91,100,121,126,237] and in rectal swabs ≥ 3 weeks in 15 cases aged 0.15–17 years [86,91,93,168,203]. In a cohort of 46 children and adolescents, four had positive rectal swabs within 2–12 days after recovery and hospital discharge with negative OP swabs [229]. An asymptomatic girl had SARS-CoV-2 RNA detectable in anal swabs for 42 days, but not in NP swabs [203].

Higher SARS-CoV-2 VL in stool than OP swabs were reported for > 20 days in an infant with mild COVID-19 [121]. Eight children had higher average VL in anal swabs compared with NP swabs [86]. Han and colleagues reported of a neonate with mild COVID-19 who had SARS-CoV-2 RNA detectable in respiratory swabs (NP swab VL: 1.2×10^10^ copies/mL; OP swab VL: 1.3×10^8^ copies/mL to undetectable 4–17 dps), stool (VL: 1.7×10^6^ copies/mL to 4.1×10^7^ copies/mL 6–18 dps), saliva (6–9 dps), plasma (5–10 dps) and urine (6–17 dps) [178].

### Immunocompromised individuals

We reviewed 33 studies including 317 immunocompromised individuals [16,50,80,88,95,96,103,110,136,147,151,152,154,155,165,169,188,191,193,207,218,219,221,233,245,249,251,253,257-261], however specifics for them were rarely outlined in cohorts. Data on SARS-CoV-2 shedding patterns in immunocompromised individuals are still limited and quantitative data are lacking. SARS-CoV-2 RNA was detected in NP swabs 57 and 63 dps in a kidney transplant recipient following clinical recovery and hospital discharge 35 dps [258]. In a report on two lung transplant recipients, an asymptomatic adolescent had a positive NP swab 26 days after diagnosis, while a mildly symptomatic adult’s NP swab was negative for SARS-CoV-2 RNA after 2 weeks [251]. In a study of 90 transplant recipients with COVID-19, seven (three with mild, four with severe disease) cases had an initial negative NP swab, but dps were not reported [207]. Evidence of endothelial cell infection and endothelitis was observed in three severe COVID-19 cases, including a renal transplant recipient [188].

A person living with human immunodeficiency virus (PLHIV) and hepatitis C virus (HCV) had pneumonia resolution and negative NP swabs 10 dps but delayed antibody response 42 dps [152]. Further three PLHIV had a similar mild course with negative NP and OP swabs after 1 week [218] and another two PLHIV after 2 weeks from diagnosis [80,165].

## Discussion and knowledge gaps

Sensitive molecular diagnostics through PCR and correct sampling and test interpretation are crucial for accurate COVID-19 diagnosis and thereby essential for good clinical practice. Furthermore, they are a key factor in outbreak phases where active case finding in combination with quarantine and contact tracing are crucial for outbreak control. We reviewed in the early stages of the pandemic knowledge on the kinetics of SARS-CoV-2 RNA shedding in different clinical samples of 32,515 COVID-19 patients to inform clinical and public health decision making.

Our review comes with limitations like the choice of rapid instead of systematic style. Given the need for timely diagnostics information in the current pandemic and the overwhelming amount of publications indexed daily, we opted for a faster adapted systematic approach. We did not assess the included publications for biases nor could fully trace for duplicate reporting of cases. Full comparability of the assays used could not be assured due to the lack of standardised diagnostic set-up across the world with respect to e.g. sampling materials, transport conditions, cell culture, extraction and PCR platforms. These were infrequently detailed in the publications. Furthermore, we did not review separately results from PCR assays targeting different parts of SARS-CoV-2 genome, for example envelope (E) [10,276,277], RNA-dependent RNA polymerase (RdRp) [9,276,277], open reading frame (ORF)1ab [11,50,51,59,276], ORF1a [276], ORF1b [276,278], nucleaocapsid (N) [11,50,51,59,276,278,279] or spike (S) [9,51,59,276,280,281] genes, nor any effect of the different platforms used, though we tried to provide the authors’ estimations of VLs or if not Ct values, where available. However, relatively few of the reviewed articles provided such detailed data, and we could not assess the data quality. We could not estimate to what extent the genetic drift in the SARS-CoV-2 genome affects the reported PCR results, nor could summarise false-positive or false-negative rates [282] due to insufficient data. Regular monitoring of the assays performance, detailed reporting and strict quality assurance mechanisms in accredited laboratories are vital to molecular diagnostics of SARS-CoV-2.

Respiratory tract specimens are the samples of choice for widespread screening and clinical course monitoring purposes, as well as for discharge and de-isolation [283,284]. Further reports with well-documented sampling time points, comparing the different types of respiratory samples, alternative sampling like oral fluid/saliva, and their diagnostic window of use, are needed. Viral RNA concentrations in the URT peak in the early infection days, including in asymptomatic and mildly symptomatic cases. SARS-CoV-2 was successfully isolated from respiratory samples with data suggesting independent replication potential in both the upper and lower respiratory tract. Thus, respiratory sampling is the optimal strategy for both symptomatic and pre-/a-symptomatic cases and for the latter should be examined in conjunction with epidemiological evidence and clinical follow-up.

Although molecular detection on URT samples is the recommended method to diagnose a SARS-CoV-2 infection, additional assays like serology are occasionally imperative to complement RT-PCR findings, e.g. in cases of insufficient clinical sensitivity of the RT-PCR over the disease course period [285]. In addition, serology can aid decision making on clinical and infection prevention management, e.g. by confirming the presence of SARS-CoV-2 specific (neutralising) antibodies in cases with consistently low VLs (high Ct values) in URT samples [286,287]. SARS-CoV-2 RNA detection in the respiratory tract during convalescence can be prolonged. However, detection of viral genomes does not directly imply the presence of infectious virus and thereby infectivity. More information on the presence of viable SARS-CoV-2 infectivity is urgently needed, especially with de-isolation strategies in mind.

Although faecal–oral transmission has not been confirmed, it cannot be firmly excluded due to evidence of SARS-CoV-2 isolation from the GI tract [58,271]. The detection of viable SARS-CoV-2 in faecal samples also has implications for the diagnostic possibilities of faeces tests. Laboratory diagnostic and safety protocols might need adjustments, for example by implementing inactivation steps and flow cabinet work. Stool and anal swabs are not samples of choice for screening purposes, though they might play a role in clinical monitoring. Some data suggested a shift from respiratory to GI shedding in the course of COVID-19 infection or independent replication in the GI tract. More information is needed on the significance of SARS-CoV-2 detection in faecal samples and how it relates to the timing of convalescence, as well as to the viral shedding specifics in children.

SARS-CoV-2 RNA in the blood has been detected mainly in cases with severe disease and blood sampling could be useful in monitoring hospitalised patients. At this stage, there is no evidence to suggest SARS-CoV-2 could be transmitted through blood. More systematic data are needed to guide blood and organ transplantation safety protocols [288].

There were single reports on viral RNA detection in samples obtained during delivery and thus questions on the use of such reproductive tract sampling, as well as on the potential for vertical or sexual transmission of SARS-CoV-2 remain. Viral RNA was detected in semen [235], but virus isolation was not attempted. Data on SARS-CoV-2 kinetics in vulnerable groups like immunocompromised patients are insufficient. Neurological signs and syndromes associated with COVID-19 and the diagnostic/monitoring potential of CSF testing remain to be clarified.

In conclusion, using SARS-CoV-2 molecular testing by amplification techniques to the maximum of its potential is a combination of choosing the most appropriate sample type, collected with adequate sampling technique, and with the infection timeline in mind. Like all diagnostic techniques, it should be analysed considering strengths and limitations, and in conjunction with the clinical presentation and epidemiological evidence. Further well-documented systematic studies are needed to characterise fully the COVID-19 clinical course and guide public health decisions concerning optimal testing strategies.

## Supplementary Data

117000 Supplementary Dataset

## Supplementary Data

343000 Supplementary Table
