# Supplementary material for: Towards a sensitive and accurate interpretation of molecular testing for SARS-CoV-2: a rapid review of 264 studies
Source: Euro Surveill. 2021 Mar 11;26(10):2001134. doi: 10.2807/1560-7917.ES.2021.26.10.2001134 (PMC7953531; doi:10.2807/1560-7917.ES.2021.26.10.2001134)
Supplement: Supplementary Table [file 2001134_REUSKEN_Supplementary_Table.pdf]

Disclaimer: This supplementary material is hosted by Eurosurveillance as supporting information alongside the article [Towards a sensitive and accurate interpretation of molecular testing for SARS-CoV-2: a rapid review of 264 studies], on behalf of the authors, who remain responsible for the accuracy and appropriateness of the content. The same standards for ethics, copyright, attributions and permissions as for the article apply. Supplements are not edited by Eurosurveillance and the journal is not responsible for the maintenance of any links or email addresses provided therein.

Supplementary Table. Characteristics of patients (n=217) with samples (n=2777) aggregated in Figure and Table

First author	Country	No of cases	Patient ID	Sex	Age	Comorbidities	Samples available	COVID-19 severity	Hospital admission dps	Hospital discharge dps
Marchand-Senécal, Xavier [1]	Canada	1		M	56		NP, OP	mild	1	9
Chen, Dabiao [2]	China	1		F	46		OP	mild	8	24
Chen, Weilie [3]	China	6	Patient 1	M	NA		OP, A, B	severe	5 transfer	17 transfer
			Patient 2	M	NA		OP, A, B	severe	5	10 transfer
			Patient 3	M	NA		OP, A, B	severe	11 transfer	
			Patient 4	M	NA		B	severe	6 tranfer	9 transfer
			Patient 5	F	NA		OP, B	severe	4	
			Patient 6	M	NA		OP, A, B	severe	5	13 transfer
Han, Wenzheng [4]	China	1		M	47	hypertension, diabetes type 2	NP	severe	7	19
Hu, Y. [5]	China	3	Case 1	F	28	NA	NP, A	mild	1	NA
		3	Case 2	M	25	NA	NP, A	mild	1	NA
		3	Case 3	M	32	NA	NP, A	mild	3	NA
Huang, Yongbo [6]	China	16	Patients#1	NA	NA		NP, OP, Sp, St, B	severe	14 ICU	56 ICU
			Patients#2	NA	NA		NP, OP, Sp, St, A, B, U	severe	12 ICU	48 ICU
			Patients#3	NA	NA		NP, OP, Sp, St, A, B, U	severe	9 ICU	47 ICU

			Patients#4	NA	NA	NP, OP, Sp, St, A, B, U	severe	9 ICU	55 ICU
			Patients#5	NA	NA	NP, OP, Sp, St, A, B, U	severe	17 ICU	54 ICU
			Patients#6	NA	NA	NP, OP, Sp, St, A, B, U	severe	11 ICU	45 ICU
			Patients#7	NA	NA	NP, OP, Sp, St, A, B, U	severe	15 ICU	
			Patients#8	NA	NA	NP, OP, Sp, St, A, B, U	severe	9 ICU	19 ICU
			Patients#9	NA	NA	NP, OP, Sp, St, A, B, U	severe	7 ICU	
			Patients#10	NA	NA	NP, OP, Sp, St, A, B, U	severe	12 ICU	31 ICU
			Patients#11	NA	NA	NP, OP, Sp, St, A, B, U	severe	14 ICU	NA
			Patients#12	NA	NA	NP, OP, Sp, St, A, B, U	severe	20 ICU	43 ICU
			Patients#13	NA	NA	NP, OP, Sp, St, B, U	severe	21 ICU	NA
			Patients#14	NA	NA	NP, OP, Sp, St, A, B, U	severe	23 ICU	NA
			Patients#15	NA	NA	NP, OP, Sp, St, A, B, U	severe	10 ICU	30 ICU
			Patients#16	NA	NA	NP, OP, Sp, St, A, B, U	severe	9 ICU	NA
Peng, Liang [7]	China	9	Patient 1	M	37	OP, A, B, U	mild	NA	NA
			Patient 2	F	46	OP, A, B, U	mild	NA	NA
			Patient 3	F	27	OP, A, B, U	mild	NA	NA
			Patient 4	F	27	OP, A, B, U	mild	NA	NA
			Patient 5	F	62	OP, A, B, U	mild	NA	NA
			Patient 6	M	30	OP, A, B, U	mild	NA	NA
			Patient 7	F	31	OP, A, B, U	mild	NA	NA

			Patient 8	M	49	hypertension and hyperthyroidism	OP, A, B, U	mild	NA	NA
			Patient 9	M	41		OP, A, B, U	mild	NA	NA
Qian, Guo-Qing [8]	China	1		M	47		OP, A	mild	9	44
Xia, Jianhua [9]	China	30	1	M	46		Sp	mild	NA	NA
			2	M	62		Sp	mild	NA	NA
			3	F	63		Sp	mild	NA	NA
			4	F	30		Sp	mild	NA	NA
			5	F	45		Sp	mild	NA	NA
			6	M	62		Sp	mild	NA	NA
			7	M	50		Sp	mild	NA	NA
			8	F	38		Sp	mild	NA	NA
			9	M	30		Sp	mild	NA	NA
			10	F	53		Sp	mild	NA	NA
			11	M	51		Sp	mild	NA	NA
			12	M	70		Sp	mild	NA	NA
			13	F	65		Sp	mild	NA	NA
			14	M	51		Sp	mild	NA	NA
			15	F	45		Sp	mild	NA	NA
			16	F	64		Sp	mild	NA	NA
			17	M	13		Sp	mild	NA	NA

			18	M	55	Sp	mild	NA	NA
			19	M	48	Sp	mild	NA	NA
			20	M	57	Sp	mild	NA	NA
			21	M	53	Sp	mild	NA	NA
			22	M	60	Sp	severe	NA	NA
			23	M	72	Sp	severe	NA	NA
			24	M	51	Sp	severe	NA	NA
			25	M	83	Sp	severe	NA	NA
			26	M	74	Sp	severe	NA	NA
			27	F	65	Sp	severe	NA	NA
			28	M	62	Sp	severe	NA	NA
			29	M	51	Sp	severe	NA	NA
			30	M	66	Sp	severe	NA	NA
Xiao, Ai Tang [10]	China	56	Patient1	M	48	NP+OP	mild	NA	NA
			Patient2	F	33	NP+OP	mild	NA	NA
			Patient3	M	39	NP+OP	mild	NA	NA
			Patient4	F	33	NP+OP	mild	NA	NA
			Patient5	M	35	NP+OP	mild	NA	NA
			Patient6	M	44	NP+OP	mild	NA	NA
			Patient7	F	47	NP+OP	mild	NA	NA
			Patient8	F	64	NP+OP	mild	NA	NA

Patient9	M	54	NP+OP	mild	NA	NA
Patient10	M	34	NP+OP	mild	NA	NA
Patient11	M	57	NP+OP	mild	NA	NA
Patient12	F	60	NP+OP	mild	NA	NA
Patient13	M	45	NP+OP	mild	NA	NA
Patient14	M	64	NP+OP	mild	NA	NA
Patient15	M	39	NP+OP	mild	NA	NA
Patient16	F	69	NP+OP	mild	NA	NA
Patient17	M	43	NP+OP	mild	NA	NA
Patient18	F	35	NP+OP	mild	NA	NA
Patient19	M	52	NP+OP	mild	NA	NA
Patient20	M	41	NP+OP	mild	NA	NA
Patient21	M	75	NP+OP	mild	NA	NA
Patient22	F	25	NP+OP	mild	NA	NA
Patient23	M	40	NP+OP	mild	NA	NA
Patient24	M	70	NP+OP	mild	NA	NA
Patient25	F	82	NP+OP	mild	NA	NA
Patient26	M	82	NP+OP	mild	NA	NA
Patient27	M	42	NP+OP	mild	NA	NA
Patient28	F	56	NP+OP	mild	NA	NA
Patient29	M	53	NP+OP	mild	NA	NA

	Patient30	F	68		NP+OP	mild	NA	NA
	Patient31	M	69		NP+OP	mild	NA	NA
	Patient32	M	34		NP+OP	mild	NA	NA
	Patient33	M	46		NP+OP	mild	NA	NA
	Patient34	M	51		NP+OP	mild	NA	NA
	Patient35	M	44		NP+OP	mild	NA	NA
	Patient36	M	57		NP+OP	mild	NA	NA
	Patient37	F	39		NP+OP	mild	NA	NA
	Patient38	F	71		NP+OP	mild	NA	NA
	Patient39	F	65		NP+OP	mild	NA	NA
	Patient40	F	33		NP+OP	mild	NA	NA
	Patient41	M	73		NP+OP	mild	NA	NA
	Patient42	F	69		NP+OP	mild	NA	NA
	Patient43	M	47		NP+OP	mild	NA	NA
	Patient44	F	81		NP+OP	mild	NA	NA
	Patient45	F	72		NP+OP	mild	NA	NA
	Patient46	M	65		NP+OP	mild	NA	NA
	Patient47	M	83		NP+OP	mild	NA	NA
	Patient48	F	58		NP+OP	mild	NA	NA
	Patient49	M	71		NP+OP	mild	NA	NA
	Patient50	F	57		NP+OP	mild	NA	NA

			Patient51	M	61		NP+OP	mild	NA	NA
			Patient52	M	42		NP+OP	mild	NA	NA
			Patient53	F	68		NP+OP	mild	NA	NA
			Patient54	F	64		NP+OP	mild	NA	NA
			Patient55	M	63		NP+OP	mild	NA	NA
			Patient56	M	43		NP+OP	mild	NA	NA
Xiao, Fei [11]	China	1		M	78		NP+OP, St, B, U	severe	8	
Xing, Yuanyuan [12]	China	2	Case 1	M	40s	NA	OP, St	mild	4	27
			Case 2	F	20s	NA	OP	mild	8	16
Zou, Lirong [13]	China	17	B	M	44		NP, OP	mild	NA	NA
			C	M	56		NP, OP	mild	NA	NA
			D	F	60		NP, OP	mild	NA	NA
			E	M	36		NP, OP	severe	NA	NA
			F	M	69		NP, OP	mild	NA	NA
			H	F	49		NP, OP	mild	NA	NA
			I	M	78		NP, OP	severe	NA	NA
			K	F	66		NP, OP	mild	NA	NA
			L	F	38		NP, OP	mild	NA	NA
			N	F	50		NP, OP	mild	NA	NA
			O	F	28		NP, OP	mild	NA	NA
			P	F	76		NP, OP	severe	NA	NA

			Q	F	70		NP, OP	mild	NA	NA
			S	M	65		NP, OP	mild	NA	NA
			T	F	58		NP, OP	mild	NA	NA
			W	M	60		OP	mild	NA	NA
			X	M	60		NP	mild	NA	NA
Haveri, Anu [14]	Finland	1		F	30s	NA	NP, B	mild	3	11
Bernard Stoecklin, Sibylle [15]	France	3	Case 1[a]	M	48		NP	severe	8	NA
		3	Case 2[a]	M	31		NP	severe	6	25
		3	Case 3[a]	F	30		NP	mild	2	21
Danis, Kostas [16]	France	4	Case 2	NA	NA	NA	NP	mild	10	21
		4	Case 3	NA	NA	NA	NP	mild	7	18
		4	Case 5	NA	NA	NA	NP	mild	11	20
		4	Case 13	NA	NA	NA	NP	mild	-4	11
Lescure, Francois-Xavier [17]	France	5	Patient 1[a]	M	31		NP, St, B, U	severe	6	25
		5	Patient 2[a]	M	48		NP, St, B	severe	8	NA
		5	Patient 3	M	80	thyroid cancer	NP, St, B, U	severe	4	24
		5	Patient 4[a]	F	30		NP, St, B, U	mild	2	21
		5	Patient 5	F	46	none	NP, St, B, U	mild	1	17
Hoehl, Sebastian [18]	Germany	1	Patient B	F	44		OP	mild	12	19

Wölfel, Roman [19]	Germany	9	#1		NA	hypothyreoidism	NP+OP, Sp, St	mild	NA	NA
			#2		NA	none	NP+OP, Sp, St	mild	NA	NA
			#3		NA	COPD	NP+OP, Sp, St	mild	NA	NA
			#4		NA	none	NP+OP, Sp, St	mild	NA	NA
			#7		NA	hyper-cholesterinemia	NP+OP, Sp, St	mild	NA	NA
			#8		NA	none	NP+OP, Sp, St	mild	NA	NA
			#10		NA	none	NP+OP, Sp, St	mild	NA	NA
			#14		NA	none	NP+OP, Sp, St	mild	NA	NA
			#16		NA	none	NP+OP, Sp, St	mild	NA	NA
Chan, Jasper Fuk-Woo [20]	Hong Kong	5	Patient 1	F	65	hypertension	NP, B, U	mild	7	NA
			Patient 2	M	66	hypertension	NP, B, U	mild	6	NA
			Patient 3	F	37	none	NP, OP, St, B, U	mild	9	NA
			Patient 4	M	36	Chronic sinusitis	NP, OP, St, B, U	mild	10	NA
			Patient 7	F	63	diabetes	NP, OP, Sp, St, B	mild	7	NA
Paoli, D. [21]	Italy	1		M	31	dyslipidemia	NP, U	mild	None	None
Arashiro, Takeshi [22]	Japan	2	Case 1	F	35	none	NP, OP	mild	6	23
			Case 2	M	27	none	NP, OP	mild	5	
Bastola, Anup [23]	Nepal	1		M	32	none	OP	mild	11	15
Seah, Ivan Yu Jun [24]	Singapore	17	1		NA		NP	mild	NA	NA
			2		NA		NP	mild	NA	9

			3	NA	NP	mild	NA	NA
			4	NA	NP	mild	NA	NA
			5	NA	NP	mild	NA	NA
			6	NA	NP	mild	NA	8
			7	NA	NP	mild	NA	15
			8	NA	NP	mild	NA	NA
			9	NA	NP	mild	NA	NA
			10	NA	NP	mild	NA	14
			11	NA	NP	mild	NA	NA
			12	NA	NP	mild	NA	25
			13	NA	NP	mild	NA	NA
			14	NA	NP	mild	NA	NA
			15	NA	NP	mild	NA	17
			16	NA	NP	mild	NA	21
			17	NA	NP	mild	NA	22
Young, Barnaby Edward [25]	Singapore	18	1	NA	NP	severe	2	29
			2	NA	NP, St, B, U	mild	3	18
			3	NA	NP	mild	7	38
			4	NA	NP	mild	3	21
			5	NA	NP, B	severe	3	25
			6	NA	NP	mild	2	26

			7		NA		NP, B, U	mild	1	12
			8		NA		NP	mild	4	22
			9		NA		NP, St, B, U	mild	5	30
			10		NA		NP, St, B, U	severe	8	19
			11		NA		NP, St, B	mild	2	14
			12		NA		NP, B, U	mild	4	17
			13		NA		NP, St, B, U	severe	1	13
			14		NA		NP, St, U	mild	2	19
			15		NA		NP, B, U	severe	1	18
			16		NA		NP	mild	7	28
			17		NA		NP, St, B, U	mild	1	12
			18		NA		NP, St, B, U	mild	2	29
Kim, Jin Yong [26]	South Korea	2	Patient 1	F	35	none	NP+OP, Sp, St, B, U	mild	2	20
			Patient 2	M	55	none	NP+OP, Sp, St, B, U	mild	15	27
Lim, Jaegyun [27]	South Korea	1	index patient	M	54	none	Sp	mild	3	19
Baettig, Sascha J [28]	Switzerland	2	patient 1	M	NA	none	NP	mild	None	None
			patient 2	M	NA	none	NP	mild	None	None
Cheng, Shao-Chung [29]	Taiwan	1		F	55	hypothyroidism	OP, Sp, St, U	mild	10	28
Winichakoon, Poramed [30]	Thailand	1		M	28	none	NP, OP	mild	3	20
Holshue, Michelle L [31]	USA	1		M	35		NP, OP, St, B, U	mild	5	

Scott, Sarah E [32]	USA	1		M	26	none	NP, OP, Sp, B	mild	None	None

A: anal/rectal swab; B: blood sample (serum, plasma, whole blood or not specified); dps: days post symptoms onset; F: female; M: male; NA: not available: NP: nasopharyngeal/midturbinate/nasal swab; NP+OP: nasopharyngeal+oropharyngeal swab (both swabs collected in one tube or results published aggregated); OP: oropharyngeal/throat swab; Sp: sputum (induced/spontaneous sputum); St: stool; U: urine.

[a] Same patient described in different publications.

Supplementary table references

1. Marchand-Senécal X, Kozak R, Mubareka S, Salt N, Gubbay JB, Eshaghi A, et al. Diagnosis and Management of First Case of COVID-19 in Canada: Lessons applied from SARS. Clin Infect Dis. 2020 Mar 9.
2. Chen D, Xu W, Lei Z, Huang Z, Liu J, Gao Z, et al. Recurrence of positive SARS-CoV-2 RNA in COVID-19: A case report. Int J Infect Dis. 2020 Mar 5;93:297-9.
3. Chen W, Lan Y, Yuan X, Deng X, Li Y, Cai X, et al. Detectable 2019-nCoV viral RNA in blood is a strong indicator for the further clinical severity. Emerg Microbes Infect. 2020;9(1):469-73.
4. Han W, Quan B, Guo W, Zhang J, Lu Y, Feng G, et al. The course of clinical diagnosis and treatment of a case infected with coronavirus disease 2019. J Med Virol. 2020 May;92(5):461-3.
5. Hu Y, Shen L, Yao Y, Xu Z, Zhou J, Zhou H. A report of three COVID-19 cases with prolonged viral RNA detection in anal swabs. Clin Microbiol Infect. 2020 2020/06/01/;26(6):786-7.
6. Huang Y, Chen S, Yang Z, Guan W, Liu D, Lin Z, et al. SARS-CoV-2 Viral Load in Clinical Samples of Critically Ill Patients. Am J Respir Crit Care Med. 2020 Apr 15.
7. Peng L, Liu J, Xu W, Luo Q, Chen D, Lei Z, et al. SARS-CoV-2 can be detected in urine, blood, anal swabs, and oropharyngeal swabs specimens. J Med Virol. 2020 Apr 24.
8. Qian G-Q, Chen X-Q, Lv D-F, Ma AHY, Wang L-P, Yang N-B, et al. Duration of SARS-CoV-2 viral shedding during COVID-19 infection. Infect Dis (Lond). 2020 Apr 10;52(7):1-2.
9. Xia J, Tong J, Liu M, Shen Y, Guo D. Evaluation of coronavirus in tears and conjunctival secretions of patients with SARS-CoV-2 infection. J Med Virol. 2020 Feb 26:10.1002/jmv.25725.
10. Xiao AT, Tong YX, Zhang S. Profile of RT-PCR for SARS-CoV-2: a preliminary study from 56 COVID-19 patients. Clin Infect Dis. 2020 Apr 19.
11. Xiao F, Tang M, Zheng X, Liu Y, Li X, Shan H. Evidence for Gastrointestinal Infection of SARS-CoV-2. Gastroenterology. 2020 2020/03/03/;158(6):1831-3 e3.
12. Xing Y, Mo P, Xiao Y, Zhao O, Zhang Y, Wang F. Post-discharge surveillance and positive virus detection in two medical staff recovered from coronavirus disease 2019 (COVID-19), China, January to February 2020. Euro Surveill. 2020 Mar;25(10):2000191.
13. Zou L, Ruan F, Huang M, Liang L, Huang H, Hong Z, et al. SARS-CoV-2 Viral Load in Upper Respiratory Specimens of Infected Patients. N Engl J Med. 2020 Mar 19;382(12):1177-9.
14. Haveri A, Smura T, Kuivanen S, Österlund P, Hepojoki J, Ikonen N, et al. Serological and molecular findings during SARS-CoV-2 infection: the first case study in Finland, January to February 2020. Euro Surveill. 2020 Mar;25(11):2000266.
15. Bernard Stoecklin S, Rolland P, Silue Y, Mailles A, Campese C, Simondon A, et al. First cases of coronavirus disease 2019 (COVID-19) in France: surveillance, investigations and control measures, January 2020. Euro Surveill. 2020 Feb;25(6):10.2807/1560-7917.ES.2020.25.6.2000094.
16. Danis K, Epaulard O, Bénet T, Gaymard A, Campoy S, Bothelo-Nevers E, et al. Cluster of coronavirus disease 2019 (Covid-19) in the French Alps, 2020. Clin Infect Dis. 2020 Apr 11.
17. Lescure F-X, Bouadma L, Nguyen D, Parisey M, Wicky P-H, Behillil S, et al. Clinical and virological data of the first cases of COVID-19 in Europe: a case series. Lancet Infect Dis. 2020 Mar 27.
18. Hoehl S, Berger A, Kortenbusch M, Cinatl J, Bojkova D, Rabenau H, et al. Evidence of SARS-CoV-2 Infection in Returning Travelers from Wuhan, China. N Engl J Med. 2020 Mar 26;382(13):1278-80.

19. Wölfel R, Corman VM, Guggemos W, Seilmaier M, Zange S, Müller MA, et al. Virological assessment of hospitalized patients with COVID-2019. Nature. 2020 Apr 1;581(7809):465-9.
20. Chan JF-W, Yuan S, Kok K-H, To KK-W, Chu H, Yang J, et al. A familial cluster of pneumonia associated with the 2019 novel coronavirus indicating person-to-person transmission: a study of a family cluster. Lancet. 2020 Feb 15;395(10223):514-23.
21. Paoli D, Pallotti F, Colangelo S, Basilico F, Mazzuti L, Turriziani O, et al. Study of SARS-CoV-2 in semen and urine samples of a volunteer with positive naso-pharyngeal swab. J Endocrinol Invest. 2020 Apr 23.
22. Arashiro T, Furukawa K, Nakamura A. COVID-19 in 2 Persons with Mild Upper Respiratory Tract Symptoms on a Cruise Ship, Japan. Emerg Infect Dis. 2020 Jun 17;26(6):10.3201/eid2606.200452.
23. Bastola A, Sah R, Rodriguez-Morales AJ, Lal BK, Jha R, Ojha HC, et al. The first 2019 novel coronavirus case in Nepal. Lancet Infect Dis. 2020 Mar;20(3):279-80.
24. Seah IYJ, Anderson DE, Kang AEZ, Wang L, Rao P, Young BE, et al. Assessing Viral Shedding and Infectivity of Tears in Coronavirus Disease 2019 (COVID-19) Patients. Ophthalmology. 2020 Mar 24.
25. Young BE, Ong SWX, Kalimuddin S, Low JG, Tan SY, Loh J, et al. Epidemiologic Features and Clinical Course of Patients Infected With SARS-CoV-2 in Singapore. JAMA. 2020 Mar 3:10.1001/jama.2020.3204.
26. Kim JY, Ko JH, Kim Y, Kim YJ, Kim JM, Chung YS, et al. Viral Load Kinetics of SARS-CoV-2 Infection in First Two Patients in Korea. J Korean Med Sci. 2020 Feb 24;35(7):e86.
27. Lim J, Jeon S, Shin HY, Kim MJ, Seong YM, Lee WJ, et al. Case of the Index Patient Who Caused Tertiary Transmission of COVID-19 Infection in Korea: the Application of Lopinavir/Ritonavir for the Treatment of COVID-19 Infected Pneumonia Monitored by Quantitative RT-PCR. J Korean Med Sci. 2020 Feb 17;35(6):e79.
28. Baettig SJ, Parini A, Cardona I, Morand GB. Case series of coronavirus (SARS-CoV-2) in a military recruit school: clinical, sanitary and logistical implications. BMJ Mil Health. 2020 Apr 16.
29. Cheng S-C, Chang Y-C, Fan Chiang Y-L, Chien Y-C, Cheng M, Yang C-H, et al. First case of Coronavirus Disease 2019 (COVID-19) pneumonia in Taiwan. J Formos Med Assoc. 2020 Mar;119(3):747-51.
30. Winichakoon P, Chaiwarith R, Liwsrisakun C, Salee P, Goonna A, Limsukon A, et al. Negative Nasopharyngeal and Oropharyngeal Swabs Do Not Rule Out COVID-19. J Clin Microbiol. 2020 Feb 26;58(5).
31. Holshue ML, DeBolt C, Lindquist S, Lofy KH, Wiesman J, Bruce H, et al. First Case of 2019 Novel Coronavirus in the United States. N Engl J Med. 2020 Mar 5;382(10):929-36.
32. Scott SE, Zabel K, Collins J, Hobbs KC, Kretschmer MJ, Lach M, et al. First Mildly Ill, Non-Hospitalized Case of Coronavirus Disease 2019 (COVID-19) Without Viral Transmission in the United States — Maricopa County, Arizona, 2020. Clin Infect Dis. 2020 Apr 2.
